# Exposure to gestational diabetes mellitus in utero impacts hippocampal functional connectivity in response to food cues in children

**DOI:** 10.1038/s41366-024-01608-1

**Published:** 2024-08-28

**Authors:** Sixiu Zhao, Lorenzo Semeia, Ralf Veit, Shan Luo, Brendan C. Angelo, Ting Chow, Andreas L. Birkenfeld, Hubert Preissl, Anny H. Xiang, Kathleen A. Page, Stephanie Kullmann

**Affiliations:** 1 Institute for Diabetes Research and Metabolic Diseases of the Helmholtz Center Munich at the University of Tübingen, Tübingen, Germany; 2 German Center for Diabetes Research (DZD), Tübingen, Germany; 3 Division of Endocrinology, Keck School of Medicine, University of Southern California, Los Angeles, CA USA; 4 Diabetes and Obesity Research Institute, Keck School of Medicine, University of Southern California, Los Angeles, CA USA; 5 Department of Psychology, University of Southern California, Los Angeles, CA USA; 6 Imaging Genetics Center, Mark and Mary Stevens Neuroimaging and Informatics Institute, Keck School of Medicine, University of Southern California, Los Angeles, CA USA; 7 Department of Research and Evaluation, Kaiser Permanente Southern California, Pasadena, CA USA; 8 Department of Internal Medicine, Division of Diabetology, Endocrinology and Nephrology, Eberhard Karls University Tübingen, Tübingen, Germany; 9 Department of Pharmacy and Biochemistry, Eberhard Karls University Tübingen, Tübingen, Germany; 10 Neuroscience Graduate Program, University of Southern California, Los Angeles, CA USA

**Keywords:** Risk factors, Obesity

## Abstract

**Objectives:**

Intrauterine exposure to gestational diabetes mellitus (GDM) increases the risk of obesity in the offspring, but little is known about the underlying neural mechanisms. The hippocampus is crucial for food intake regulation and is vulnerable to the effects of obesity. The purpose of the study was to investigate whether GDM exposure affects hippocampal functional connectivity during exposure to food cues using functional magnetic resonance imaging (fMRI).

**Methods:**

Participants were 90 children age 7–11 years (53 females) who underwent an fMRI-based visual food cue task in the fasted state. Hippocampal functional connectivity (FC) was examined using generalized psychophysiological interaction in response to food versus non-food cues. Hippocampal FC was compared between children with and without GDM exposure, while controlling for possible confounding effects of age, sex and waist-to-hip ratio. In addition, the influence of childhood and maternal obesity were investigated using multiple regression models.

**Results:**

While viewing high caloric food cues compared to non-food cure, children with GDM exposure exhibited higher hippocampal FC to the insula and striatum (i.e., putamen, pallidum and nucleus accumbens) compared to unexposed children. With increasing BMI, children with GDM exposure had lower hippocampal FC to the somatosensory cortex (i.e., postcentral gyrus).

**Conclusions:**

Intrauterine exposure to GDM was associated with higher food-cue induced hippocampal FC especially to reward processing regions. Future studies with longitudinal measurements are needed to clarify whether altered hippocampal FC may raise the risk of the development of metabolic diseases later in life.

## Introduction

Gestational diabetes mellitus (GDM) is traditionally defined as glucose intolerance with first-time diagnosis during pregnancy [1]. It develops in approximately 10% of pregnancies, making it one of the prevalent complications during gestation [2]. Intrauterine exposure to GDM increases the risk of developing obesity in offspring [2]. It is not yet clear which factors might drive these conditions later in life, but early neurodevelopmental processes appear sensitive to intrauterine hyperglycemia, hyperinsulinemia and neuroinflammation caused by maternal overnutrition, including hyperglycemia [3, 4]. Furthermore, intrauterine exposure to GDM may lead to increased food intake, which is regulated by multiple brain regions, as the hypothalamus, striatum, insula, hippocampus etc. [5, 6]. Significantly, functional imaging data demonstrated that food cue reactivity in these brain regions can predict weight gain including in children [7, 8].

Children exposed to GDM display higher food cue reactivity in the orbitofrontal cortex [9], fail to inhibit hypothalamic activity after glucose ingestion [10] and exhibit hypothalamic inflammation [11]. Moreover, data from animals and humans suggests the development of the hippocampus is sensitive to adverse in utero environmental exposures (e.g., GDM) [4, 12–15]. In animals, intrauterine exposure to diabetes caused decreased neuronal density and reduced synaptic integrity in the hippocampus [4, 12, 13]. GDM exposure in utero and maternal obesity also associated with reduced thickness and volume in the hippocampus in children [14, 15].

The hippocampus is known for its major role in learning and memory and is believed to influence food intake by integrating learned experiences with interoceptive signals (for review, see [16]). Animal models and behavioral studies in humans suggest that even a brief exposure to a diet rich in dietary fat and sugar can impair hippocampal-dependent learning and memory [17, 18]. Behavioral data in healthy humans showed that influencing meal memory can reduce or enhance later food intake [19, 20]. Furthermore, amnesic patients fail to interpret interoceptive signals related to hunger and satiety [21]. Using fMRI, the hippocampus has been shown to be responsive to the ingestion of sugar, visual food cues, and postprandial hormones in healthy adults [16, 22, 23]. Hence, hippocampal dysfunctions may impair the ability to retrieve memories of meals, detect interoceptive signals, which may lead to overeating (for reviews, see [24]).

However, there is currently no available research on the hippocampus functional network in response to visual food cues in children with GDM exposure, who exhibit higher risk of developing obesity [2]. Thus, the current study investigates the relation between GDM exposure and functional connectivity (FC) of the hippocampus in children.

We examined task-based FC of the bilateral hippocampus in children with and without GDM exposure using generalized psychophysiological interaction (gPPI) in response to visual food cues (food minus non-food) in the BrainChild Cohort [9, 25]. Prior studies [26–32] indicate higher food-cue-induced neural reactivity of reward regions and alterations in hippocampal FC in both children and adults with obesity. Hence, we hypothesized that hippocampal FC is higher to reward-related regions during food cue presentation in children with GDM exposure when compared to children without exposure. In addition, we explored the relationship between adiposity measures of children and mothers and hippocampal FC. Given prior evidence suggesting that GDM has distinct effects on the left and right hippocampus in children [14], we conducted separate exploratory analyses on the FC of the left and right hippocampus.

## Methods

### Participants

Participants included 112 children from the larger BrainChild study assessing the impact of exposure to GDM in utero on neural and endocrine systems underlying risk for obesity and diabetes [10]. The BrainChild study included typically developing children aged 7–11 years recruited from Kaiser Permanente Southern California (KPSC) [9, 25]. Inclusion criteria included KPSC’s electronic medical records, which documented maternal GDM or normal glucose tolerance during pregnancy, uncomplicated singleton birth, and children with no history of medical/psychiatric disorders or taking medicines affecting metabolism. Twenty-two participants were excluded due to excessive movement, image artifacts, or the presence of brain lesions. The final analyses included a total of 90 participants. Based on the sample size of *N* = 90 and the detected effect size of 0.8 (primary analysis: GDM versus Non-GDM), we achieved a statistical power of 0.96 at an alpha level of 0.05.

### Ethics approval and consent to participate

The institutional review board at both KPSC (# 10282) and University of Southern California (USC) (# HS-14-00034) approved this study. This study was in accordance with the Declaration of Helsinki. Parents and children were provided with written informed consent and informed child assent prior to the study.

### Maternal GDM exposure

GDM during pregnancy was determined based on one of the following laboratory plasma glucose values during pregnancy: (1) plasma glucose values ≥ 200 mg/dL from a 50 g 1-hr glucose challenge test, (2) at least two plasma glucose values meeting or exceeding the following values on either the 75 g 2-hrs or 100 g 3-hrs oral glucose tolerance test: fasting, 95 mg/dL; 1 h, 180 mg/dL; 2 h, 155 mg/dL; and 3 h, 140 mg/dL [33].

### Study procedures

The data for this study were collected over two visits conducted after a 12-h overnight fast. The first visit consisted of metabolic phenotyping, including assessments of anthropometric measures. The second visit was a neuroimaging visit, including functional magnetic resonance imaging (fMRI) measurement during a food cue task after the overnight fast.

### First visit: anthropometric measurement

During the first visit, anthropometric data, including height, weight, waist and hip circumferences of both the mother and child, tanner stage of child were collected at the Clinical Research Unit of the USC Diabetes and Obesity Research Institute as previously reported [10]. Specific to children, BMI *z*-scores (BMI-z) were calculated using the Center for Disease Control (CDC) guidelines [34].

### Second visit: MRI measurement

After the overnight fast, fMRI measurements of the children were performed at the USC Dana and David Dornsife Neuroimaging Center. Children first underwent training on a mock scanner, after which they were imaged in a 3 T MRI scanner. All children were scanned between 8 and 10 am following 12-h of overnight fasting. They completed a visual food cue task in the scanner (For more details, see [25]). Briefly, children were presented high-calorie food (e.g., ice cream) and non-food (e.g., pencils) pictures and instructed to watch the pictures attentively. The stimuli were selected based on pilot studies of children’s ratings of familiarity and appeal of the food and non-food items. And, the food and non-food tems were also selected to include similar characteristics such as contrast, salience, color, shape and complexity. A total of 12 blocks of stimuli were included, comprising an equal distribution of 50% food images and 50% non-food images. Each block included three images and each image was displayed for 4 s with 1 s consistent inter-stimulus interval between pictures. The sequence of the blocks was randomized. The food cue task lasted 196 s in total. The task was designed to be particularly efficient for differential effects (food versus non-food) with a short stimulus onset asynchrony and not for common task effects or task effects versus implicit baseline.

### Image acquisition and preprocessing

The imaging was conducted on a Siemens MAGNETOM Prismafit 3 T MRI scanner with a 20-channel head coil. Functional images were obtained using a 2D single-shot gradient echo planar imaging sequence with the following parameters: repetition time (TR) = 2000 ms; echo time = 25 ms; flip angle = 85°; voxel resolution 3.4 × 3.4 × 4 mm^3^; 32 axial slices. A high-resolution structural image was also acquired at 1 × 1 × 1 mm^3^ resolution. For more details, see publication [25].

The preprocessing of the fMRI data was performed using SPM12 (http://www.fil.ion.ucl.ac.uk/spm). Slice timing and realignment were performed for each fMRI time series. Movement criteria was movement > 2° or 2 mm in any direction, or mean framewise displacement of more than 0.3 mm. The resulting mean functional image and the structural image was coregistered. Unified segmentation was performed to the anatomical image and normalization parameters were estimated. Then, these parameters were applied to the functional images and normalized into Montreal Neurological Institute (MNI) space, using the same method applied in our previous paper by Luo et al. [25] and in other studies [35, 36] with children within the same age range. The data were then smoothed with an 8 mm field-width half-maximum (FWHM) Gaussian kernel. Physiological noise signals in the white matter and cerebrospinal fluid were extracted using Principal Component Analysis (PCA) using the PhysIO toolbox [37].

### Region of interest (ROI) definition

**Fig. 1:**
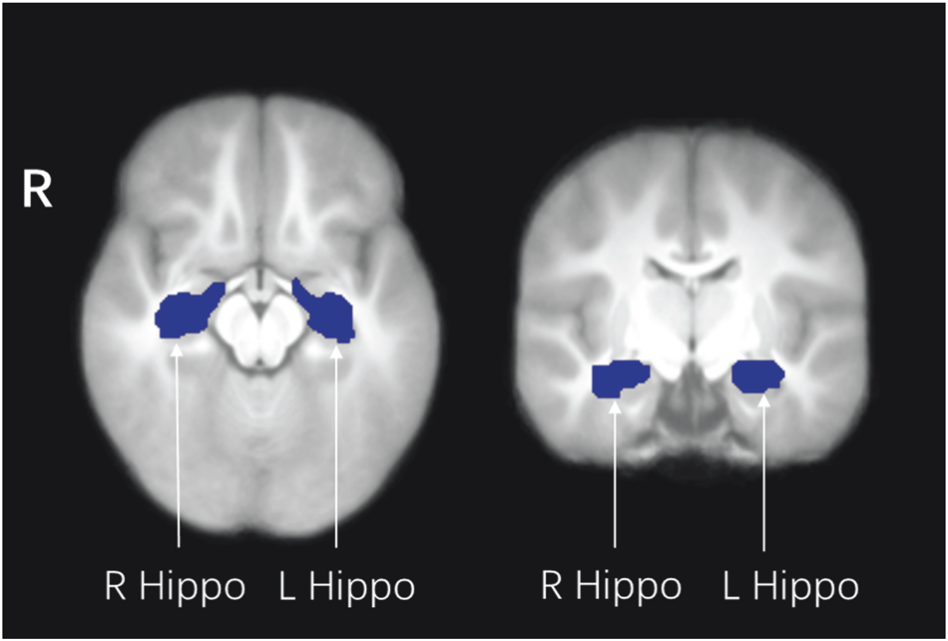
Masks of the hippocampus derived from the AAL atlas 3, overlaid on the average normalized T1 weighted image of the children. Hippo, Hippocampus; L, left; R, right.

To specifically investigate the effect of GDM on the hippocampus FC, we used an anatomical ROI-based approach. Left, right and bilateral ROIs of the hippocampus were created using the AAL atlas 3 (AAL3, https://www.oxcns.org) (Fig. 1).

### Generalized psychophysiological interaction (first level analysis)

For each participant, the brain response to high-calorie food and non-food images was convolved with a canonical hemodynamic response function, and then added to the General Linear Model (GLM). The six motion parameters, and three components each of the white matter and cerebrospinal fluid signals extracted by PCA were also included in the GLM as confounds. High-pass filtering was applied using bandwidth = 0.0078 (1/128) Hz.

Task-based FC between anatomical seed region of the hippocampus (i.e., bilateral hippocampus) and all other brain voxels was assessed using a generalized psychophysiological interaction (gPPI) approach (https://www.nitrc.org/projects/gppi version 13.1). In an exploratory analysis, FC was assessed for the right and left hippocampus separately in the same way.

First, the time series from the seed region were extracted. Second, the PPI interaction terms were generated for food and non-food stimuli according to the time series. Finally, FC of the seed region was computed for food and non-food stimuli for each participant.

### Statistical analyses

#### Hippocampal functional connectivity in response to food minus non-food cues

To evaluate intrauterine exposure to GDM on food-cue induced hippocampal FC, the gPPI contrast maps of *food minus non-food* were entered into a second-level two-sample t-test model with the GDM exposure (GDM vs. Non-GDM) as grouping factor. Age and sex were included in the model as covariates due to their potential effects on hippocampal structure and function [14, 38]. Waist-to-hip ratio (WHR) rather than BMI has been reported to be positively correlated with hippocampus activity in response to food cues [39] and we recently reported higher WHR in children with GDM exposure [9]. Therefore, WHR was adjusted for the possible impact of adiposity.

The statistical parametric maps were thresholded using an uncorrected threshold of *p* < 0.001 and a cluster-level family-wise error (FWE) corrected threshold of *p* < 0.05. In addition, small volume correction (SVC) was performed for the insula and striatum (caudate, putamen, nucleus accumbens, pallidum), based on their activation in response to food reward processing and influenced by obesity in children and adolescents [40, 41]. The striatal mask and the insular mask were generated based on AAL3 (https://www.oxcns.org) and the wfu pick atlas (https://www.nitrc.org/projects/wfu_pickatlas/). Multiple comparison was implemented for two masks using corrected threshold *p* < 0.025.

#### Correlation between task-based hippocampal functional connectivity and obesity measures of children and mothers

To explore the effect of children’s obesity and maternal adiposity on bilateral hippocampal FC in children, a second-level multiple regression model was created using SPM 12 at the whole-brain level. This analysis was performed separately for children with and without GDM exposure. These models included the gPPI *food minus non-food* contrast as intercept, with WHR, BMI *z*-score, maternal current BMI or maternal prepregnancy BMI as the regressors of interest, adjusted for age and sex. An uncorrected threshold of *p* < 0.001 and a cluster-level FWE corrected threshold of *p* < 0.05 were used. The correlations were assessed for the right and left hippocampus separately in the same way.

## Results

### Demographics

The demographics of the 90 participants included in this study are shown in Table 1 (ages 7–11 years, 53 females, 50 GDM exposed), and 89% of children were in Tanner Stage 1. There were no significant differences in children’s age, sex, BMI *z*-score, or maternal current BMI or maternal prepregnancy BMI among GDM exposed vs. unexposed groups (*p* > 0.05, Table 1). There was a trend towards a higher WHR for children exposed to GDM than unexposed (t [88] = 1.97, *p* = 0.052, Table 1).

**Table 1:** Demographics*.

	Overall	Non-GDM	GDM	t/Z	*p*
	90	*N* = 40	*N* = 50		
**Children**					
Age (years)	8.23 (7.82, 9.08)	8.61 (7.75, 9.67)	8.14 (7.85, 8.63)	1.406	0.30
Sex					0.13
Female	53 (58.9%)	20 (50%)	33 (66%)		
Male	37 (41.1%)	20 (50%)	17 (34%)		
BMI *z*-score	0.66 (0.03, 1.67)	0.59 (-0.06, 1.68)	0.76 (0.23, 1.69)	0.285	0.43
WHR	0.87 ± 0.06	0.86 ± 0.06	0.88 ± 0.06	1.969	0.052
**Mother**					
Current BMI (kg/m^2^)	30.27 (26.52, 35.43)	29.58 (25.60, 35.06)	30.71 (26.90, 36.11)	4.921	0.64
Prepregnancy BMI (kg/m^2^)	29.07 (25.23, 33.22)	29.07 (24.61, 32.98)	28.98 (25.37, 33.51)	2.186	0.61

*BMI* body mass index, *GDM* gestational diabetes mellitus, *WHR* waist-to-hip ratio, *t* statistic for two-sided independent-samples *t*-test, *Z* statistic for Mann–Whitney test for data with skewed distribution.

*For continuous variables, normally distributed data (WHR) were described as mean ± standard deviation (SD); data from skewed distribution were described by the median (Q1, Q3); Categorical variable was described as *N* (%), *p* value was calculated using Chi-square test.

### Hippocampal functional connectivity in response to food minus non-food cues

**Fig. 2:**
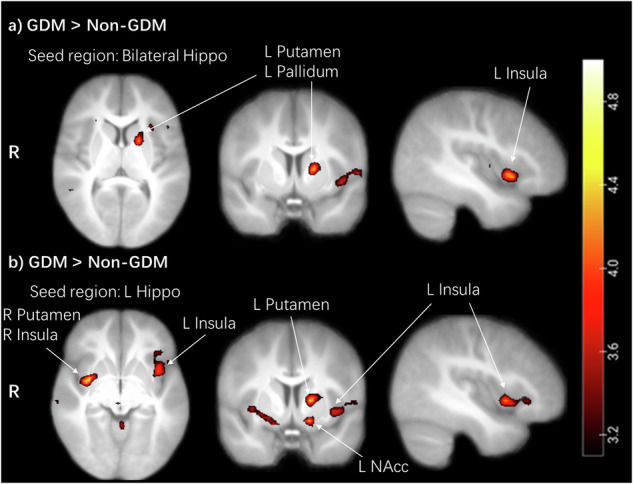
Hippocampal functional connectivity during the food-cue task. **a** Children with GDM exposure showed higher FC between bilateral hippocampus and left insula, left putamen/pallidum. **b** Children with GDM exposure showed higher FC between left hippocampus and the bilateral putamen, insula, and left NAcc. The cluster of the right putamen extended to the right insula. The cluster of left putamen extended to the left pallidum. Color map corresponds to T values (*p* < 0.001 uncorrected for display) overlaid on the normalized average T1 weighted image of the children. Hippo hippocampus, FC functional connectivity, GDM gestational diabetes mellitus, NAcc nucleus accumbens, L left, R right.

We observed higher FC in children with GDM exposure compared to children without GDM exposure between the bilateral hippocampus and the left insula (*p*_FWE_ = 0.037) and left putamen, which extended to the left pallidum (*p*_FWE_ = 0.019, SVC) (Table 2, Fig. 2).

In an exploratory analysis, FC was assessed for the right and left hippocampus separately. In children with GDM exposure compared to children without exposure, we observed higher FC between the left hippocampus and the right putamen (*p*_FWE_ = 0.007), left putamen (*p*_FWE_ = 0.017, SVC), right insula (*p*_FWE_ = 0.017), left insula (*p*_FWE_ = 0.011, SVC), and left nucleus accumbens (NAcc, *p*_FWE_ = 0.013, SVC) (Table 2, Fig. 2). The cluster of the right putamen extended to the right insula. The cluster of the left putamen extended to the left pallidum. No group differences were found for the right hippocampus.

**Table 2:** Hippocampus task-based functional connectivity in response to food versus non-food cues adjusted for age, sex and WHR.

	Brain region	Hemi	MNI coordinates			Peak t	Cluster size	*p*_FWE_
			x	y	z			
Seed region	GDM > Non-GDM							
Bilateral Hippo								
	Insula	L	−42	14	−7	4.31	59	0.037
	Pallidum/ Putamen	L	−15	2	2	4.29	23	0.019^SVC^
Hippo L								
	Putamen	R	36	−1	−4	4.87	91	0.007
	Insula	R	39	−1	−4	4.20	74	0.017
	NAcc	L	−15	5	−13	4.41	5	0.013^SVC^
	Putamen/Pallidum	L	−18	5	5	4.32	30	0.017^SVC^
	Insula	L	−42	8	−4	4.42	20	0.011^SVC^
Hippo R								
	No differential activation							
	Non-GDM > GDM							
	No differential activation							

*FWE* family wise error, *GDM* gestational diabetes mellitus, *Hemi* hemisphere, *NAcc* nucleus accumbens, *WHR* waist-to-hip ratio, *L* left, *R* right, *p* value FWE corrected using whole-brain cluster correction, *SVC* *p*_FWE_ small volume corrected for ROIs.

### Association between task-based hippocampal functional connectivity and obesity measures of children and mothers

No significant correlation was observed between the FC of the bilateral hippocampus and WHR, BMI *z*-score, maternal current or maternal prepregnancy BMI in both the GDM and Non-GDM groups (all *p*_FWE-corrected_ > 0.05).

**Fig. 3:**
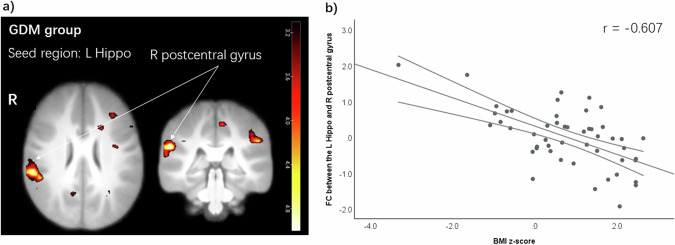
Hippocampal functional connectivity in relation to BMI *z*-score in the GDM group. **a** Children with GDM exposure showed lower FC between the left hippocampus and the right postcentral gyrus with higher BMI *z*-score. Color map corresponds to *T* values (Multiple regression analysis with BMI *z*-score; *p* < 0.001 uncorrected for display) overlaid on the normalized average T1 weighted image of the children. **b** Negative correlation between BMI *z*-score and the extracted cluster of the FC of the left hippocampus and the right postcentral gyrus in the GDM group. Error bars indicate 95% confidence interval. Hippo hippocampus, FC functional connectivity, GDM gestational diabetes mellitus, L left, R right.

Further analysis of the FC of the left or right hippocampus separately revealed significant correlations. In the GDM group, there was a negative correlation between BMI *z*-score and the FC of the left hippocampus and the right postcentral gyrus (peak-voxel (MNI) x: 57, y: −34, z: 26); *r* = −0.607; *p*_FWE-corrected_ < 0.001) (Fig. 3). In the Non-GDM group, a positive correlation was found between the maternal current BMI and the FC of the left hippocampus to the right superior frontal gyrus (peak-voxel (MNI) x: 18, y: 59, z: −1); *r* = 0.574; *p*_FWE-corrected_ = 0.001).

## Discussion

The current study investigated the relationship between intrauterine GDM exposure and food cue induced hippocampal functional connectivity in children aged 7–11 years in the fasted state. Consistent with our hypothesis, children with GDM exposure compared to unexposed showed higher hippocampal FC to reward processing regions (i.e., putamen, pallidum, NAcc and insular cortex) and lower hippocampal FC to the somatosensory cortex with increasing BMI.

We observed higher functional coupling between hippocampus and striatal regions and insula in children with intrauterine GDM exposure compared to children without exposure, primarily driven by the left hippocampus. A previous structural MRI report found reduced left hippocampal thickness in children with GDM exposure compared to unexposed children [14]. Therefore, GDM may affect both the structure and function of the hippocampus.

Hippocampal neurons interact with other neurons in the mesolimbic system receiving dopamine projections to communicate rewarding properties of environmental stimuli [16, 42]. As potent rewards, palatable foods can trigger associations with reward and motivational behaviors that potentially could lead to overeating and, eventually, weight gain [42]. These food cues tend to evoke heightened memories and mental simulations of consumption in children [43]. Moreover, a meta-analysis indicated that the hippocampus-striatum connection may play a role in craving and the formation of habits associated with obesity [44]. Concomitantly, higher activation in the striatum and insula in response to food images were observed in children and adolescents with obesity compared to their healthy-weight peers [40, 41, 45]. In the resting state, higher striatal and insular network FC was also linked to eating in the absence of hunger, food craving, disinhibited eating, weight gain and obesity in both children and adults [46–49]. In the current study, no significant influence of WHR or BMI was identified on these hippocampal connections in children. However, BMI negatively correlated with the left hippocampus to the somatosensory cortex FC in children exposed to GDM, aligning with resting-state studies in children with obesity [50]. The oral somatosensory cortex is known to sense fat and food texture [51] and children and adolescents with obesity show greater activation in the somatosensory cortex to food [8, 52]. The higher preference for high-fat foods in children is a predictor of future weight gain [53]. Nonetheless, it is yet unknown whether altered hippocampal to somatosensory connectivity patterns in children with GDM exposure predict the development of obesity later in life.

Our study points to a distinct effect of intrauterine GDM exposure on the hippocampal network primarily to reward processing regions, rather than obesity itself at this young age. These results align with animal studies [4, 12, 13] and provide evidence to support the hypothesis that prenatal exposure to diabetes might result in changes in brain pathways. These changes, in turn, may contribute to the increased risk of weight gain and obesity in affected children at a later age. Interestingly, previous studies suggest that hyperactivity in the brain’s reward system might be a susceptibility factor for weight gain [8, 54]. Similarly, our previous study showed that children exposed to GDM had higher daily energy intake [9]. Moreover, parental obesity has been related to greater striatum and insula activation in response to food rewards and higher ad libitum intake even in adolescents of healthy-weight [8, 55]. In the current study, we also found higher food-cue induced hippocampal FC to the frontal cortex in children of mothers with higher BMI. Although this connection in relation to maternal obesity has not yet been fully investigated, higher FC between temporal and frontal regions has been reported in adolescent obesity [56]. Future studies with longitudinal measurements are necessary to evaluate whether hippocampal changes in FC result in weight gain and raise the risk of developing obesity later in life.

Our study includes some limitations. Given the limited size of our sample, each subgroup, based on GDM exposure, included a relatively small number of subjects. In addition, food intake and behavioral assessments were not assessed, and future studies are necessary to provide a more detailed understanding how the observed functional alterations in the hippocampus are related to cognitive and metabolic processes. Moreover, longitudinal data are needed to examine the association between functional alterations in the hippocampus and future weight gain in children.

## Conclusion

Our study suggests that intrauterine exposure to GDM alters hippocampal food cue processing network in children. During palatable food picture presentation, children with GDM exposure exhibited higher hippocampal connectivity specifically to reward processing regions and lower hippocampal connectivity, with increasing BMI, to the somatosensory cortex. These alterations may be associated with a potential risk for future weight gain. Longitudinal research is required to determine if altered hippocampal functional connectivity during exposure to food cues leads to future weight gain and a higher likelihood of metabolic disorders, including obesity.

## Data Availability

Data is available upon reasonable request from KAP.
